# A Review of Curcumin and Its Derivatives as Anticancer Agents

**DOI:** 10.3390/ijms20051033

**Published:** 2019-02-27

**Authors:** Mhd Anas Tomeh, Roja Hadianamrei, Xiubo Zhao

**Affiliations:** 1 Department of Chemical and Biological Engineering, University of Sheffield, Sheffield S1 3JD, UK; matomeh1@sheffield.ac.uk (M.A.T.); rhadianamrei1@sheffield.ac.uk (R.H.); 2 School of Pharmaceutical Engineering and Life Science, Changzhou University, Changzhou 213164, China

**Keywords:** curcumin, anticancer, structure activity relationship, cellular pathway, mechanism of action, delivery system

## Abstract

Cancer is the second leading cause of death in the world and one of the major public health problems. Despite the great advances in cancer therapy, the incidence and mortality rates of cancer remain high. Therefore, the quest for more efficient and less toxic cancer treatment strategies is still at the forefront of current research. Curcumin, the active ingredient of the *Curcuma longa* plant, has received great attention over the past two decades as an antioxidant, anti-inflammatory, and anticancer agent. In this review, a summary of the medicinal chemistry and pharmacology of curcumin and its derivatives in regard to anticancer activity, their main mechanisms of action, and cellular targets has been provided based on the literature data from the experimental and clinical evaluation of curcumin in cancer cell lines, animal models, and human subjects. In addition, the recent advances in the drug delivery systems for curcumin delivery to cancer cells have been highlighted.

## 1. Introduction

Cancer is the second most life-threatening disease and one of the main public health problems worldwide. In 2018, there were around 1.73 million new cases of cancer and more than 609,000 deaths in the United States alone [1]. Despite the tangible advances in cancer therapy, the reported incidence of the disease and the mortality have not declined in the past 30 years [2]. Understanding the molecular alterations that contribute to cancer development and progression is a key factor in cancer prevention and treatment. There are several common strategies for targeting specific cancer cells to inhibit tumor development, progression, and metastasis without causing severe side effects [3]. In addition to the chemically synthesized anticancer agents, several anticancer compounds with different modes of action have been extracted from plant sources, such as *Taxus brevifolia*, *Catharanthus roseus*, *Betula alba*, *Cephalotaxus* species, *Erythroxylum previllei*, *Curcuma longa*, and many others [4]. Among them, curcumin is the most important component of the rhizomes of *Curcuma longa* L. (turmeric) [5] and was extracted from turmeric plant in a pure crystalline form for the first time in 1870 [6]. Curcumin and its derivatives have received immense attention in the past two decades due to their biofunctional properties such as anti-tumor, antioxidant, and anti-inflammatory activities [7]. These properties are attributed to the key elements in the curcumin structure [8]. Therefore, a great deal of scientific work has shed light on the structure activity relationship (SAR) of curcumin in an attempt to improve its physiochemical and biological properties. Due to the importance of cancer as a leading cause of death and the ongoing quest for more efficient and less toxic anticancer agents, this review has mainly focused on the anticancer activity of curcumin. The applications of curcumin in other diseases are beyond the scope of this review and have been reviewed elsewhere [4,9].

The main mechanisms of action by which curcumin exhibits its unique anticancer activity include inducing apoptosis and inhibiting proliferation and invasion of tumors by suppressing a variety of cellular signaling pathways [10]. Several studies reported curcumin’s antitumor activity on breast cancer, lung cancer, head and neck squamous cell carcinoma, prostate cancer, and brain tumors [11], showing its capability to target multiple cancer cell lines. In spite of all the above mentioned advantages, curcumin’s applications are limited due to its low water solubility which results in poor oral bioavailability and also low chemical stability [7]. Another obstacle is the low cellular uptake of curcumin. Due to its hydrophobicity, the curcumin molecule tends to penetrate into the cell membrane and bind to the fatty acyl chains of membrane lipids through hydrogen binding and hydrophobic interactions, resulting in low availability of curcumin inside the cytoplasm [12,13]. To overcome these obstacles and improve the overall anticancer activity of curcumin, several structural modifications have been suggested to enhance selective toxicity towards specific cancer cells [14], increase bioavailability, or enhance stability [4,15]. Another approach is to use different delivery systems to improve curcumin’s physiochemical properties and anticancer activity. This review focuses on the recent literature on the SAR of curcumin and its analogues and their anticancer activity in different cancer cell lines, animal models, and human clinical trials as well as different types of curcumin delivery systems that have been used for cancer therapy.

## 2. Structure Activity Relationship of Curcumin and Its Derivatives

**Figure 1:**
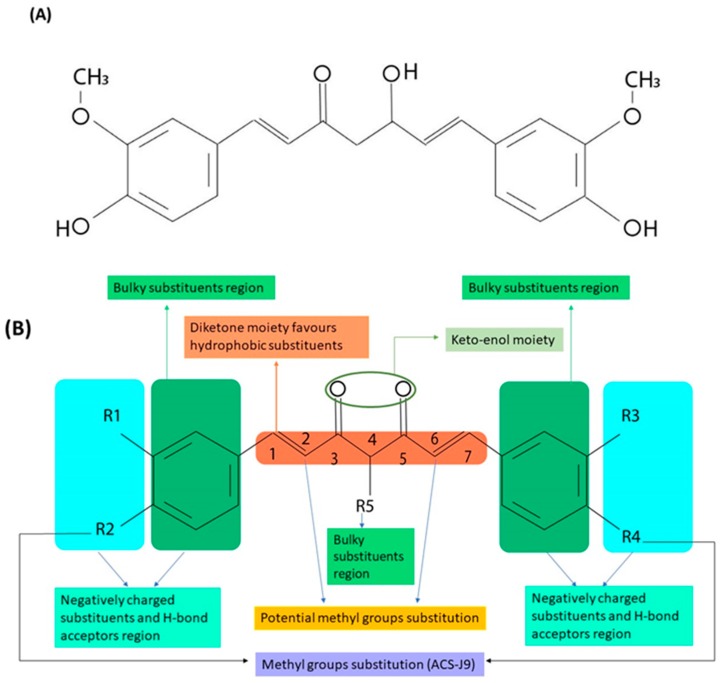
(**A**) Chemical structure of curcumin. (**B**) The main pharmacophores and potential substitution positions.

Chemical structure modification does not only affect the receptor binding and pharmacological activity of a drug molecule but also alters its pharmacokinetics and physiochemical properties [4]. Determining the essential pharmacophores within a drug molecule requires a thorough study of its natural and synthetic analogues [11]. The chemical structure of curcumin is depicted in Figure 1A. As can be observed, it consists of two phenyl rings substituted with hydroxyl and methoxyl groups and connected via a seven carbon keto-enol linker (C7). While curcumin is naturally derived, its derivatives are generally produced by a chemical reaction between aryl-aldehydes and acetylacetone. This assembly method can yield multiple chemical analogues, such as compounds with alkyl substituents on the middle carbon of the linker (C7 moiety) [16,17]. A SAR study of curcumin derivatives demonstrates that the presence of a coplanar hydrogen donor group and a β-diketone moiety is essential for the antiandrogenic activity for the treatment of prostate cancer [17]. In addition, scanning 50 curcumin analogues showed that shortening the linker from seven carbon atoms (C7) to five carbon atoms (C5) improves the antiandrogenic activity [18]. As a result of introducing a methyl group at both C2 and C6 positions, a new curcumin derivative has been produced (Figure 1B). This derivative exhibited a steric hindrance effect toward metabolizing enzymes, such as alcohol dehydrogenase [14], and demonstrated significantly higher activity than curcumin in inhibiting endothelial cell proliferation and invasion both in vitro and in vivo [14]. Dimethylcurcumin or ASC-J9 (5-hydroxy-1, 7-bis (3, 4-dimethoxyphenyl)-1, 4, 6-heptatrien-3-one) is a newly developed curcumin analogue which enhances androgen receptor degradation and has been used for treatment of prostate cancer [19,20,21]. Moreover, it has also shown a significant antiproliferative effect against estrogen-dependent breast cancer cells [22]. Although methylation has enhanced the targetability and activity of the molecule, it has also increased its hydrophobicity massively compared to curcumin, which has limited its administrable dose in cancer therapy [23]. 

Furthermore, studies on the kinetic stability of synthetic curcumin derivatives have pointed out that glycosylation of the pharmacophore aromatic ring improves the compound’s water solubility, which enhances its kinetic stability and leads to a better overall therapeutic response [24]. During phase Ι and phase ΙΙ metabolism, the main routes of converting curcumin into a higher excretable form are oxidation, reduction, and conjugation (glucuronidation and sulfurylation). The conjugation reactions occur on the hydroxyl groups (4-OH) attached to the phenyl rings of curcumin. Thus, curcumin’s kinetic stability can be enhanced by masking the 4-OH groups [25]. Another study has revealed a correlation between the hydrophobic property of the benzyl rings and androgen receptor affinity [26]. The benzyl rings are also crucial for inhibiting tumor growth, and adding hydrophobic substituents, such as CH_3_ groups, on them (R1, R2, R3, R4 in Figure 1B) have been linked to the increased antitumor activity of curcumin derivatives [26,27]. O-methoxy substitution was found to be more effective in suppressing nuclear factor κ-light-chain-enhancer of activated B cells (NF-κB), but this modification has also affected the lipophilicity of curcumin [28]. A summary of the potential sites of modification on the curcumin molecule is illustrated in Figure 1B.

Some of the modified curcuminoids exhibit enhanced anticancer and anti-inflammatory activities compared to curcumin due to the low level of hydrogenation, high level of methoxylation, and unsaturation of the diketone moiety [29]. Ortho-methoxy substitution of the essential radical of curcuminoid alters the heptadiene moiety and the hydrogenation level [28]. A comparative study on curcumin and its derivatives revealed stronger antioxidant activity for several hydrogenated curcumin derivatives compared to the original curcumin compound [30]. For example, tetrahydrocurcumin (THC) exhibited higher antioxidant activity than dihydrocurcumin (DHC) and unmodified curcumin [31,32] (Table 1). Unlike curcumin, THC, which is a non-electrophilic derivative, failed to suppress the signal transducer and activator of transcription 3 (STAT3) signaling pathway and induce apoptosis. This suggests that the electrophilic nature of curcumin is essential for inhibiting the STAT3 signaling pathway during anticancer therapy [33]. Metallo-curcumin-conjugated DNA complexes have been constructed using Cu^2+^/Ni^2+^/Zn^2+^ metal ions to improve curcumin solubility and enhance DNA-binding ability [34]. These complexes also showed a better antibacterial activity and significant toxicity to several prostate cancer cell lines [34] (Table 1).

In addition to anticancer and anti-inflammatory properties, curcuminoids exert antioxidant activity mainly through the chelating effect of the diketone moiety. The presence of metals such as Cu^2+^, Fe^2+^, and Pb^2+^ boost the chelating power of curcumin derivatives [35]. The unsaturated diketone group in curcumin root is a Michael reaction acceptor, part of phase ΙΙ enzyme inducers [4], which can be responsible for NF-κB suppression in cancer cells. However, an investigation on 72 different curcumin derivatives did not find a direct correlation between the inhibition of tumor growth through NF-κB and antioxidant activity [36]. *O*-methoxy substitution resulted in increased antioxidant activity of curcumin only when the methoxy group was not linked to the proton acceptor β-diketone moiety through conjugation [4]. The equilibrium between the keto and enol forms of curcumin relies on environmental factors such as pH. The keto form is dominant at acidic or neutral pH, while the enol form is more common in basic pH [37]. This unique property has been exploited in the discovery of new curcumin nanoassemblies with a buffering capacity that exhibit the “proton sponge effect” in endosomes and lysosomes [7]. 

Although curcumin has low water solubility and poor bioavailability, it enjoys a strong pharmacological effect in clinical applications [48]. A novel study attempted to explain this unique property of curcumin by testing the pharmacological effect of curcumin’s metabolites resulting from physiological degradation. The parallel docking calculations of curcumin degradation products were found to be similar to those of curcumin because they share with the original compound the same binding pockets required for inhibiting several enzymes [48].

## 3. Different Types of Curcumin Delivery Systems Used in Cancer Therapy

**Figure 2:**
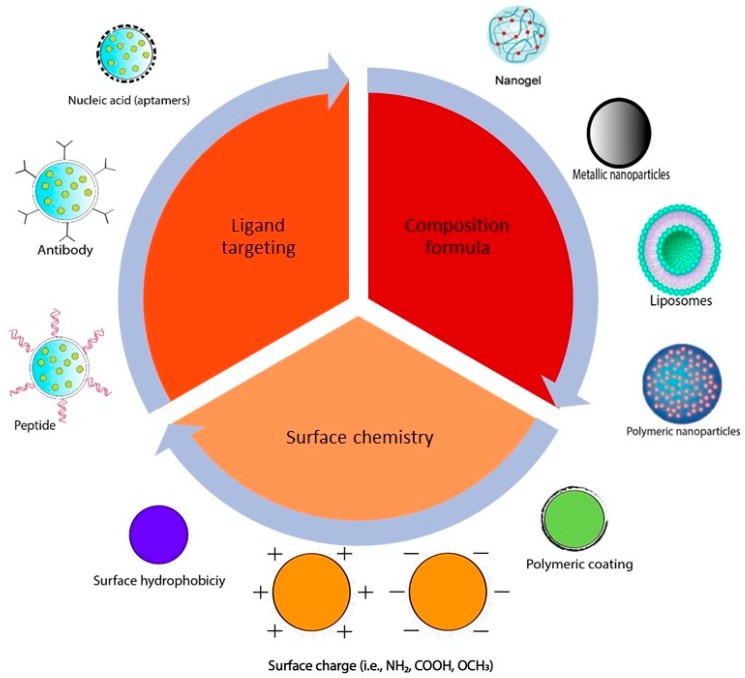
Examples of current nanoparticle design strategies used to improve targeting.

Various delivery systems for curcumin have been formulated using different nanotechnologies in order to improve curcumin properties and targetability. For the rational design of the nanoformulations, several factors should be considered in order to enhance the efficacy and improve the cellular targeting of the anticancer agents. These factors include the nanoparticle size and shape, surface properties, and nanoparticle targeting ligands [49], as illustrated in Figure 2. A summary of the most commonly used curcumin delivery systems is introduced in this section.

### 3.1. Polymeric Nanoparticles

Various polymers have been utilized to prepare nanoformulations for curcumin drug delivery to improve its biological activity [50]. The biocompatible and biodegradable polymers are preferred in the drug delivery systems due to lower risk of toxicity [51]. Therefore, biodegradable synthetic polymers such as PLGA (poly (D, L-lactic-co-glycolic acid) and natural polymers such as silk fibroin and chitosan have become widely used in drug delivery [52,53,54]. PLGA-curcumin nanoformulation was found to be as effective as curcumin at 15-fold lower concentration in inhibiting mRNAs for inflammatory cytokines (CXCR3 and CXCL10) and increasing anti-inflammatory cytokine interleukin-10 (IL-10) in the brain [55]. In vivo study in rats showed that the bioavailability of curcumin-PLGA nanospheres was increased nine-fold in comparison to unprocessed curcumin administrated with alkaloid compound piperine. However, curcumin/piperine coadministration enhanced curcumin activity by inhibiting hepatic and intestinal deactivation [56]. Another study compared the anticancer activity of curcumin-loaded PLGA nanoparticles (CUR-NPs) and curcumin-loaded PLGA nanoparticles conjugated to anti-P-glycoprotein (P-gp) (CUR-NPs-APgp). The latter formulation showed significantly more specific binding to cervical cancer cells KB-3-1 but lower entrapment efficiency compared to CUR-NPs [57]. Spherical PLGA nanospheres were also developed to encapsulate dimethyl curcumin (ASC-J9) and tested in breast cancer cells. The PLGA nanospheres were capable of releasing ASC-J9 intracellularly, leading to growth inhibition of estrogen-dependent MCF-7 cancer cells [22].

### 3.2. Liposomes

**Figure 3:**
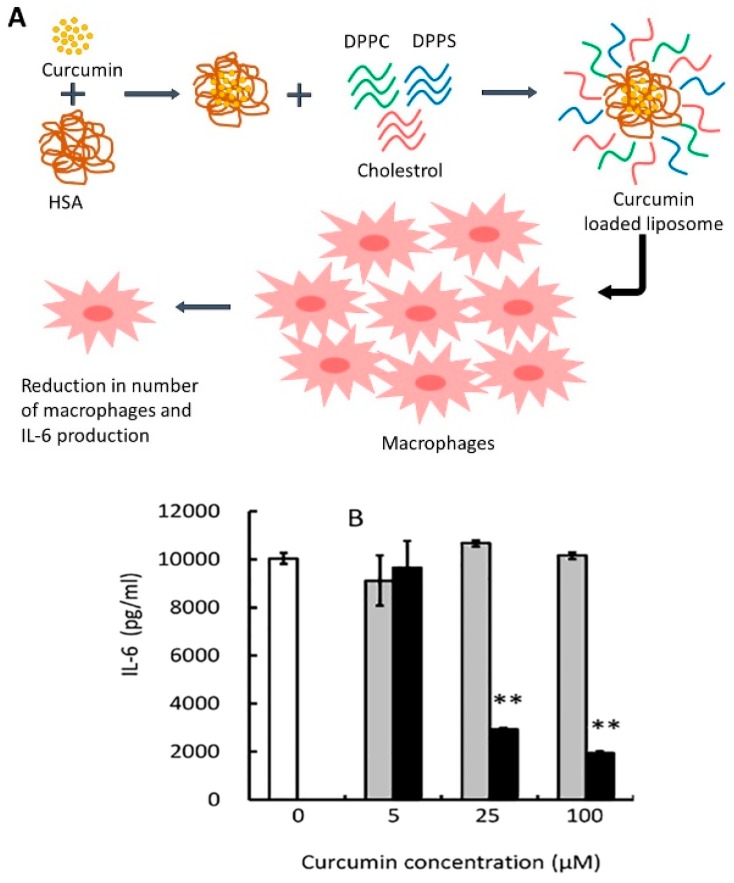
(**A**) Schematic representation of curcumin-loaded liposomes inducing a reduction in the number of macrophages [62]. HSA: human serum albumin; DPPC: 1, 2-dipalmitoyl-sn-glycero-3-phosphocholine; DPPS: 1, 2-dipalmitoyl-sn-glycero-3-phospho-L-serine. (**B**) curcumin-loaded liposomes inhibit production of IL-6; white, grey, and black columns represent control, unloaded liposomes, and curcumin-loaded liposomes respectively. Reprinted from Amano et al. [62].

Nanoscale liposomes are emerging as one of the most useful drug delivery systems for anticancer agents. Recent advances in liposome formulations have resulted in improved treatment for drug-resistant tumors and reduced toxicity [58]. A liposome consists of a phospholipid bilayer shell and an aqueous core which makes it an ideal carrier for encapsulating both hydrophobic and hydrophilic compounds. Several liposome preparations have been utilized to encapsulate curcumin (Table 2). The liposomal lipid bilayer (such as egg yolk phosphatidyl choline (EYPC), dihexyl phosphate (DHP) and cholesterol) solubilizes curcumin. This preparation was found to stabilize loaded curcumin proportionally to its content [59]. Another work on liposomes tested coating liposomes with lipid–polymer conjugate N-dodecyl chitosan-N-[(2-hydroxy-3-trimethylamine) propyl] (HPTMA) chloride. Positively charged nanoliposomes for curcumin delivery have also been developed by incorporating polyethylene glycol (PEG) and cationic polyethyleneimine (PEI) into the formulation. Despite low encapsulation efficiency (45%), this formulation has demonstrated twenty-fold higher cytotoxic activity than unprocessed curcumin in various cell lines, including human HepG2 hepatocellular carcinoma, A549 lung carcinoma, HT29 colorectal carcinoma, and cervical carcinoma [60]. In liposomal gene delivery, an interesting work conducted by Fujita et al. [61] utilized curcumin to control siRNA release. By incorporating curcumin into the liposomal formula, siRNA release showed a bell-shaped pattern due to the dose-dependent increase in liposomal permeability induced by curcumin. Curcumin-loaded liposomes were also used to inhibit the production of IL-6 in macrophages. The liposomes were prepared by mixing curcumin solution with human serum albumin (HSA) solution and subsequently adding this mixture to a lipid mixture containing 1, 2-dipalmitoyl-sn-glycero-3-phosphocholine (DPPC), 1, 2-dipalmitoyl-sn-glycero-3-phospho-L-serine sodium salt (DPPS) and cholesterol. The designed system induced significant IL-6 suppression and reduction in the total number of macrophages [62] (Figure 3).

### 3.3. Nanogels

Although hydrogels and nanogels have gained considerable attention in the past decade as a promising drug delivery system, only a few studies have investigated the curcumin-nanogel delivery in cancer therapy. There are several polymeric hydrogel nanoparticle systems that have been prepared recently using synthetic or natural polymers. Among the natural polymers, chitosan, chitin, and alginate are the most studied for the preparation of nanogels in drug delivery [73]. On the other hand, the most commonly used synthetic polymers are polyvinyl alcohol (PVA), polyethylene oxide (PEO), polyethyleneimine (PEI), polyvinyl pyrrolidone (PVP), and poly-N-isopropylacrylamide (PNIAA) [74]. One of the main advantages of natural hydrogels over synthetic ones when used in drug delivery is biodegradability and biocompatibility [70,74]. Additionally, nanogels possess unique features, including large surface area for drug entrapment and a porous structure for drug loading and release [70,75]. A curcumin-loaded chitin nanogel has been used as a transdermal system for the treatment of skin cancer [70] (Table 2) and has shown more specific toxicity towards human skin melanoma (A375) in comparison to human dermal fibroblast (HDF) cells without compromising the antitumor activity of curcumin [70]. In another study, a hybrid nanogel system consisting of alginate, chitosan, and pluronic polymers was prepared via the polycationic crosslinking method and tested on a HeLa cell line [76]. This delivery system demonstrated very high entrapment efficiency, and a significant difference in cell proliferation was observed between the cells treated with unprocessed curcumin and the cells treated with curcumin-loaded hybrid nanogel [76]. 

### 3.4. Peptide and Protein Formulations

**Figure 4:**
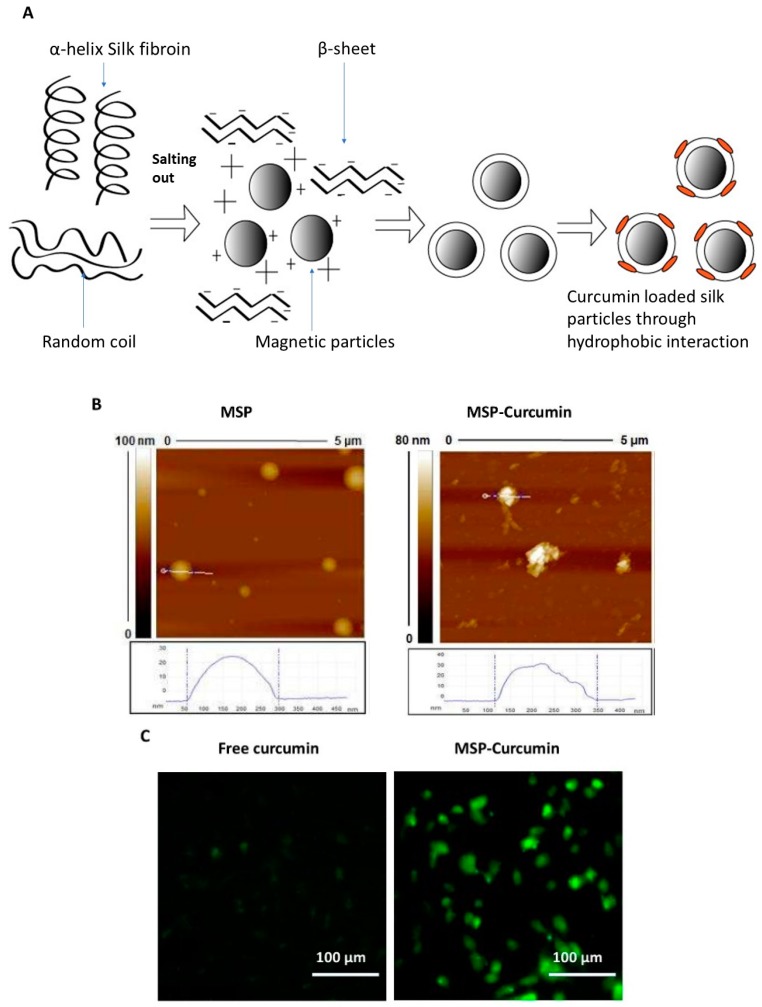
(**A**) Fabrication of magnetic silk particles (MSP) for curcumin delivery. (**B**) Atomic force microscopy (AFM) images of MSP before and after curcumin loading. (**C**) Representative microscopic images of MDA-MB-231 cells incubated with free curcumin and curcumin-loaded MSP showing a significant improvement of curcumin cellular uptake. Reprinted from Song et al. [54], copyright © 2017 ACS.

As discussed earlier, hydrogels and polymeric materials have shown promising results in curcumin drug delivery. However, a few limitations have arisen in processing clinical applications, including toxicity of unreacted monomers, post-crosslinking shrinkage or fragility of the polymer gels, and rapid discharge of a large amount of the loaded drug during the initial burst release in drug carrier [77]. In an attempt to address these limitations, self-assembling peptide systems have been developed. Peptides provide several benefits when introduced to drug delivery systems, such as biocompatibility, desirable hydrophilicity, and mild processing conditions [78]. A recent study investigated the physical properties and therapeutic efficacy of a curcumin-loaded, self-assembling (MAX8) peptide (β-hairpin) hydrogel system. This newly developed system has combined multiple advantages such as enhanced delivery, curcumin stabilization, and controlled drug release by changing the MAX8 peptide concentration [79]. In another example, an amphiphilic polypeptide (β-casein) was able to self-assemble into micelles. Encapsulation of curcumin within the hydrophobic core of the β-casein micelles increased its aqueous solubility by 2500 times [80]. Human serum albumin (HSA) is one of the most commonly used proteins in nanoparticle preparation due to its excellent biocompatibility [81]. Curcumin-loaded HSA nanoparticles have been produced through the homogenization of aqueous HSA solution (to crosslink the HSA molecules) and curcumin dissolved in chloroform. This formulation improved the curcumin solubility by 300 times but only achieved 7.2% curcumin loading efficiency, which was likely due to entrapment of curcumin within the albumin hydrophobic cavity through hydrophobic interactions [82]. Recently, silk fibroin (SF) protein has attracted tremendous attention due to its excellent biocompatibility and multiple biomedical applications [83]. Since its approval by the FDA, several studies have investigated its potential applications in drug delivery [84]. Magnetic silk nanoparticles (MSPs) were used to deliver curcumin to MDA-MB-231 breast cancer cells. As illustrated in Figure 4, these particles were fabricated using the salting-out method to convert silk from its α-helix form to the β-sheet (the insoluble) form, thus providing a hydrophobic surface for curcumin loading. The designed nanoformulation managed to achieve a small particle size (100–350 nm), cell internalization, and the possibility for additional targeting using an external magnetic field on the target tissue [54]. 

### 3.5. Cyclodextrin Complexes

**Figure 5:**
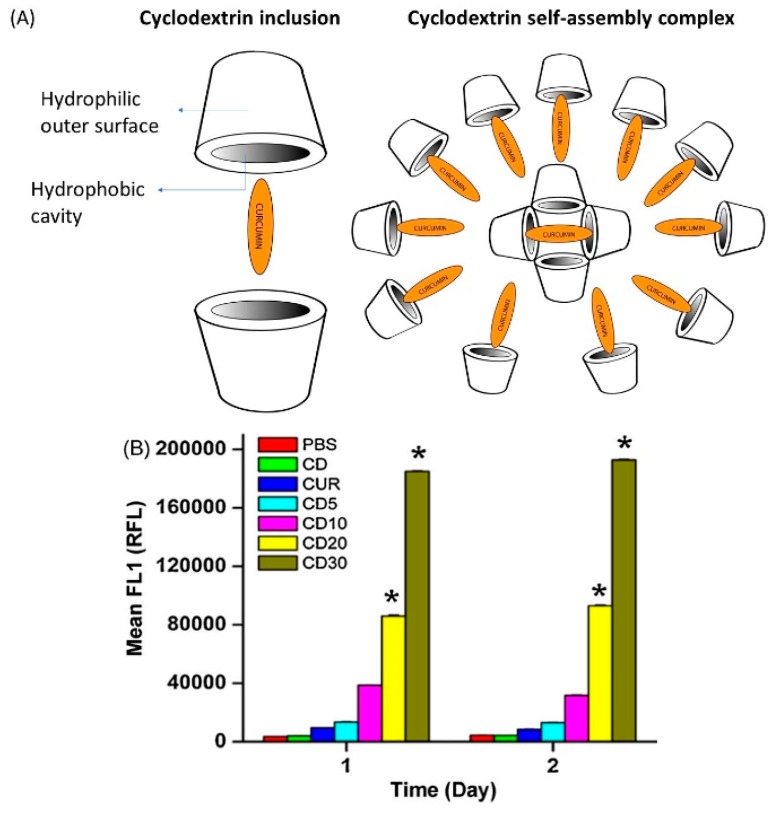
(**A**) Schematic structure of cyclodextrin–curcumin (CD–CUR) inclusion and self-assembled complexes (**B**) Fluorescence-activated cell sorting (FACS) analysis for cellular uptake of curcumin and different CD–CUR (CD5, CD10, CD20, and CD30) inclusion complexes treated in DU145 prostate cancer. * *p* < 0.05 represents significant difference from the curcumin uptake. Reprinted from Yallapu et al. [86] with permission from the copyright holder Elsevier.

Cyclodextrins are cyclic oligosaccharides which consist of a hydrophilic outer layer and a lipophilic core. In drug delivery, these complexes provide several beneficial properties, including enhanced solubility, increased bioavailability, and improved stability of the loaded drug. There are different types of cyclodextrins, such as natural (α, β, and γ), chemically modified, and polymerized cyclodextrins, that vary in water solubility and molecular weight [85]. There are also different cyclodextrin complexes, including inclusion complexes and self-assembled cyclodextrins (Figure 5A). Few studies have used cyclodextrins as carriers in curcumin delivery to enhance bioavailability, minimize degradation, and reduce nonselective toxicity [86]. A β-cyclodextrin–curcumin self-assembling preparation has shown higher uptake of curcumin by DU145 prostate cancer cells compared to unprocessed curcumin [86] As shown in Figure 5B, a significant increase in cellular uptake of cyclodextrin–curcumin (CD–CUR) inclusion complexes (CD5, CD10, CD20, and CD30) by cancer cells was observed compared to free curcumin. Another study found a complementary therapeutic effect of curcumin–cyclodextrin complexes in lung cancer. Administration of these complexes to mice with orthotopically implanted lung tumors resulted in improved bioavailability of curcumin and a significant reduction in tumor size [87]. 

## 4. Anticancer Activity of Curcumin

**Figure 6:**
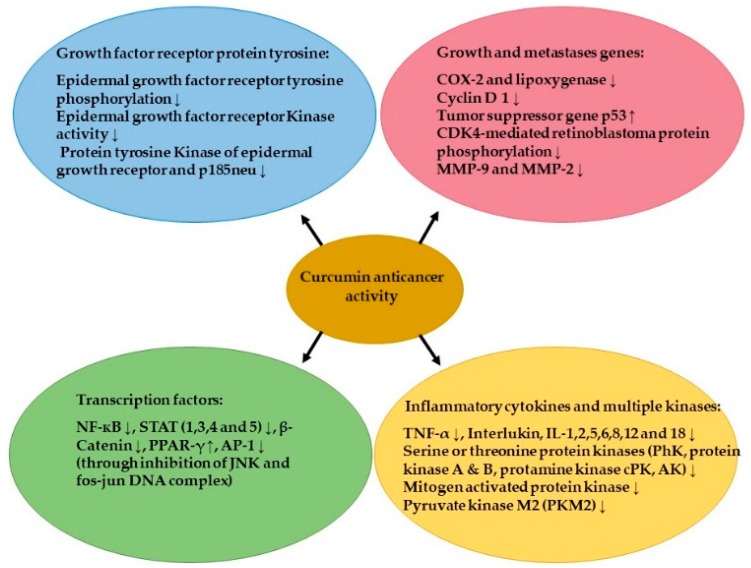
The main molecular targets of curcumin in cancer cells. ↑: Increase; ↓: Decrease; MMP: Matrix metalloproteinase; AP-1: Activation protein-1.

One of the main causes of cancer is the loss of balance between cell proliferation and cell death [88]. When the cells skip death due to the absence of the apoptotic signals, uncontrolled cell proliferation occurs, leading to different types of cancer [89]. The apoptotic signals are generated through two major pathways: the intrinsic pathway and the extrinsic pathway. The intrinsic pathway works through stimulating the mitochondrial membrane to inhibit expression of antiapoptotic proteins Bcl-2 and Bcl-Xl [90]. Curcumin disturbs the balance in the mitochondrial membrane potential, leading to enhanced suppression of the Bcl-xL protein [91]. The extrinsic apoptotic pathway works through increasing the death receptors (DRs) on cells and triggering the tumor necrosis factor (TNF)-related apoptosis. Curcumin also contributes to this pathway by upregulating the expression of death receptors DR 4 and DR 5 [92,93,94]. In vitro studies showed a remarkable ability of curcumin and its derivatives to induce apoptosis in different cell lines by inhibiting or downregulating intracellular transcription factors. These factors include NF-κB, activator protein 1 (AP-1), cyclooxygenase II (COX-2), nitric oxide synthase, matrix metalloproteinase-9 (MMP-9), and STAT3 [33,73]. A recent work has found a new anticancer mechanism for curcumin by decreasing the glucose uptake and lactate production (Warburg effect) in cancer cells via downregulation of pyruvate kinase M2 (PKM2). The inhibition of PKM2 was achieved by suppressing the mammalian target of rapamycin-hypoxia-inducible factor 1α (TOR-HIF1α) [95]. Several studies have investigated the ability of curcumin and its derivatives to suppress multiple different carcinomas by interacting with different molecular targets (Figure 6).

### 4.1. In Vitro and In Vivo Studies 

Curcumin has shown very promising results in suppressing cancer cell growth and proliferation in several different types of cancer, such as prostate, colorectal, breast, pancreatic, brain, head, and neck cancers. What comes next is a summary of the anticancer activity of curcumin and its derivatives in different types of cancer based on the data from in vitro studies in different cancer cell lines and animal studies.

#### 4.1.1. Prostate Cancer 

A recent estimate reported by the American Cancer Society revealed that 2.9 million men have been diagnosed with prostate cancer (PCa) in the United States [20], making it the second leading cause of cancer death in men [96]. Curcumin has shown a strong ability to inhibit proliferation and induce apoptosis in prostate cancer both in vitro and in vivo [97] by interfering with a number of cellular pathways, including mitogen-activated protein kinase (MAPK), epidermal growth factor receptor (EGFR), and nuclear factor κ (NFκB) [98,99]. A recent study has revealed the ability of curcumin to activate protein kinase D1 (PKD1), leading to attenuation of the oncogenic signaling by β-catenin and MAPK [100] and consequent inhibition of prostate cancer [100]. Moreover, PKD1 was found to be severely downregulated following progression from androgen-dependent to androgen-independent prostate cancer [100], and to affect the motility and invasion of prostate cancer via interaction with E-cadherin [101]. Therefore, it has been considered as a new therapeutic target for cancer in general and for prostate cancer in particular [102]. In addition to curcumin, some of its derivatives have also shown anticancer activity against prostate cancer. Metallo-curcumin conjugated DNA complexes exhibited significant toxicity to prostate cancer cells (PC3, 22Rv1, TRAMP-C1, LNCaP, and DU145) [34]. Dimethyl curcumin (ASC-J9) has also shown very good activity in enhancing androgen receptor degradation in androgen-dependent prostate cancer [20,38].

#### 4.1.2. Colorectal Cancer

Colorectal cancer comes third behind prostate cancer and lung cancer as the most common form of malignant cancer [103]. Although patients diagnosed with colorectal carcinoma undertake surgical removal of the tumor tissue along with chemotherapy, more than half of the patients suffer from relapses [104]. Administration of curcumin was found to reduce M (1) G levels in the malignant colorectal cells without changing COX-2 protein levels [105]. In addition, curcumin treatment was able to downregulate miR-21 gene, which is overexpressed in colorectal cancer cells, by inhibiting AP-1 (activator protein) binding to miR-21 promoter [101]. Treating HCT 116 colorectal cancer cells with curcumin resulted in a cell cycle arrest in the G_2_/M phase via miR-21 gene regulation and inhibited the tumor tissue growth [101]. However, an in vivo study in mice with colorectal cancer demonstrated an improved response to radiation therapy when combined with curcumin due to its ability to target nuclear factor (NF-κB) [106]. Another study has managed to enhance curcumin inhibition activity against colon cancer cells by combining it with ERRP, a pan-erb B inhibitor [107].

#### 4.1.3. Head and Neck Squamous Cell Carcinoma 

Head and neck squamous cell carcinoma (HNSCC) is the sixth most common form of cancer worldwide, with more than 30,000 diagnosed cases every year [108]. HNSCC generally arises in the oral cavity, paranasal cavities, larynx, and pharynx [10]. In vitro studies of curcumin in different head and neck cancer cell lines have proven its ability to inhibit cell growth due to its effects on a number of cellular pathways involved in cell proliferation, most notably NF-κB and STAT3, which are found to be overexpressed in several head and neck carcinomas [109,110]. Curcumin was shown to downregulate NF-κB and inhibit the interleukin-6 (IL-6)-mediated phosphorylation of STAT3, thus inhibiting the proliferation of the cancer cells [110,111]. 

#### 4.1.4. Breast Cancer

Breast cancer has shown an alarming record as a leading cause of death in women [112]. Despite lumpectomy, radiation therapy, chemotherapy, and endocrine therapy, the recurrence rate of breast cancer has been reported to be still high based on a meta-analysis of 21 retrospective studies [113]. Therefore, there is still a need for more efficient therapeutic strategies. In a study on MCF-10A human mammary epithelial cells and MCF-7 breast cancer cells [114], a tangible drop in telomerase activity was observed as a result of treatment with curcumin in a concentration-dependent manner which was correlated to downregulation of hTERT by curcumin but not through the c-Myc mRNA pathway [114]. The effect of curcumin on cell-cycle regulatory proteins, matrix metalloproteinases (MMPs), and NF-κB was evaluated in MDA-MB-231 and BT-483 breast cancer cell lines [115]. In agreement with the previous studies on other breast cancer cell lines, this study also confirmed the ability of curcumin to downregulate NF-κB, leading to an antiproliferative effect [115,116]. However, a decrease in cyclic D1 in MDA-MB-231 cells and a decrease in CDK4 BT-483 were observed after treatment with curcumin [115]. Combining arabinogalactan and curcumin enhanced apoptosis induction by increasing ROS levels, disturbing the mitochondrial membrane and decreasing glutathione in MDA-MB-231 cell line [117]. Moreover, curcumin led to the inhibition of breast tumor via overexpression of the *p53* gene and reduction of antigen ki-67 levels [117]. Another study on MDA-MB-231 cells has shown that curcumin also inhibits inflammatory cytokines CXCL1/2. Inhibiting CXCL1 and 2 by curcumin results in inhibiting the expression of a series of metastasis-promoting genes such as chemotactic receptor CXCR4 [118,119]. Dimethyl curcumin (ASC-J9) has also been reported to be effective against estrogen-dependent breast cancer via inhibiting several types of steroid receptors [22,120].

#### 4.1.5. Brain Cancer and Glioblastoma

The incidence rate of central nervous system (CNS) tumors, including brain tumors, are predicted to increase by 6% in the UK between 2014 and 2035 [121]. Glioblastoma (GBM), which is the most common malignant brain cancer in humans, accounts for about 15% of all CNS tumors [122,123]. In the treatment of brain tumors and GBM, surgical intervention and radiation therapy are limited due to infiltration of cancer cells into the healthy brain, leading to damaging effects after treatment [124]. Therefore, alternative therapies using naturally derived compounds such as curcumin with less side effects than the conventional treatments are receiving more attention. Curcumin has multiple molecular targets (Figure 6), therefore, combating brain tumors may take different cellular pathways, including apoptosis, autophagy, angiogenesis, invasion, and metastasis [123]. Although penetrating the blood–brain barrier (BBB) is considered the rate-limiting step for many anticancer agents, curcumin was able to cross the BBB in high levels [125]. Moreover, an in vivo study using human glioma U-87 cells xenografted into athymic mice showed that curcumin is able to suppress glioma angiogenesis through inhibiting MMP-9 and downregulating endothelial cell markers (CD31 and CD105 mRNA) [125]. Curcumin was also able to induce G2/M cell cycle arrest by increasing protein kinase 1 (DAPK1) in U-251 malignant glioblastoma cells, which indicates that suppressing DAPK1 by curcumin does not only induce cell arrest but also inhibits STAT3 and NF-κB and activates caspase-3 [126].

### 4.2. Clinical Studies

In addition to the studies carried out in human cell cultures or in animal models, there have been several clinical studies carried out in human subjects to evaluate the efficacy and safety of treatment with curcumin in different types of cancer either alone or in combination with other chemotherapy agents. A summary of an excerpt of these clinical studies is provided in Table 3.

#### 4.2.1. Colorectal Cancer

The pharmacology of curcumin in humans was studied in a dose-escalation study by Sharma et al. [131] on fifteen patients with histologically proven advanced adenocarcinoma of the colon or rectum refractory to standard chemotherapies. The patients received doses of curcumin between 0.45 and 3.6 g per day orally for up to four months. Subsequently, levels of curcumin and its metabolites in plasma, urine, and feces were measured. In addition, glutathione S-transferase (GST) activity, levels of oxidative DNA adduct (M_1_G), and the extent of ex vivo induction of prostaglandin E2 (PGE2) in patient blood leukocytes were measured as biomarkers of curcumin activity. Intact curcumin and its glucuronide and sulfate conjugates were detected in plasma at a concentration of 10 nmol/L and also in urine. No dose-limiting toxicity was observed. No effect on basal PGE2 levels in leukocytes was observed after administration of curcumin at any of the doses, nor were there any changes in the lipopolysaccharide (LPS)-induced production of PGE2 at doses between 0.45 and 1.8 g per day. However, administration of 3.6 g curcumin per day led to 62% and 57% reductions in the inducible PGE2 levels in patient blood samples 1 h after administration on days 1 and 29, respectively, compared to the baseline levels. Total GST activity and M_1_G levels in leukocytes showed considerable differences between patients, but no treatment-related effects were observed. Based on these results, they suggested a daily oral dose of 3.6 g of curcumin for a Phase II trial in cancers in sites outside the gastrointestinal tract which require systemic effects [131].

The pharmacological activity of curcumin in the colorectum was also studied by Garcea et al. [105] as measured by the levels of M_1_G and COX-2 in 12 patients with colorectal carcinoma following oral administration of curcumin at doses of 450 mg, 1800 mg, or 3600 mg per day. Blood samples and biopsy samples of the normal and malignant colorectal tissue were taken from the patients at designated time points and analyzed for the levels of curcumin, curcumin metabolites (curcumin sulfate and curcumin glucuronide), M_1_G, and COX-2. Higher concentrations of curcumin were observed in normal compared to malignant colorectal tissues of patients receiving 3.6 g/day of curcumin, with trace levels of curcumin in the peripheral blood circulation. Curcumin metabolites were also detected in the colorectum of these patients. On the other hand, baseline M_1_G levels were 2.5-fold higher in malignant tissue as compared with normal tissue in the same group of patients, which were reduced significantly after the administration of curcumin. Nevertheless, the levels of COX-2 in malignant colorectal tissue were not reduced by curcumin. Based on these findings, they suggested that a daily dose of 3.6 g of curcumin can reach pharmacologically active concentrations in the colorectum with minimal distribution outside the gastrointestinal system [105].

The mechanism of anticancer activity of curcumin in colorectal cancer was investigated by a number of researchers. In 2001, Plummer et al. [130] conducted a dose-escalation pilot study of the effects of Curcuma extract (containing curcumin and desmethoxycurcumin) on the inhibition of COX-2 activity and consequently the levels of PGE2 in 15 patients with advanced colorectal cancer. The patients were divided into five groups receiving doses between 40 and 200 mg of curcuminoids once per day via the oral route for a minimum of 29 days. Comparison of the PGE2 levels in blood samples from patients showed a significant difference between patients in different groups and decreased levels of PGE2 with an increased dose of curcumin, which clearly indicates dose-dependent inhibition of COX-2 by curcumin [130]. 

This was further investigated by Carroll et al. [133] who carried out a nonrandomized, open-label clinical trial to assess the effects of oral curcumin in prevention of colorectal cancer. In the study, 44 smokers with eight or more aberrant crypt foci (ACF) on screening colonoscopy were included and were divided into two groups receiving either 2 g or 4 g of curcumin per day via the oral route for 30 days. The levels of PGE2 and 5-hydroxyeicosatetraenoic acid (5-HETE) within ACF were assessed, as well as the reduction in the number and/or proliferation of ACF (measured by rectal endoscopy and Ki-67 immunohistochemistry assay, respectively). ACF reduction was used as a measure of the cancer preventive efficacy of curcumin, assuming that reducing the concentrations of PGE2 and 5-HETE in the colorectal mucosa would result in reduced epithelial crypt proliferation and ACF formation.

No reduction in the levels of PGE2 or 5-HETE within ACF or normal mucosa was observed with any doses of curcumin, nor was there any reduction in the levels of Ki-67 in normal mucosa. By the same way, there were no changes in the number of ACF in the group treated with 2 g of curcumin. However, a significant reduction in the number of ACF was observed in the group treated with 4 g of curcumin, which was associated with a significant increase in the plasma levels of curcumin conjugates, indicating the effect of systematically delivered curcumin conjugates on the reduction of ACF number, rather than locally delivered curcumin [133].

He et al. [132] investigated the effects of curcumin on the expression of *p53* in the colorectum tissue and the serum levels of TNF-α in patients with colorectal cancer. A total of 126 patients diagnosed with colorectal cancer were randomly divided into two groups receiving either curcumin (360 mg three times per day per oral route) or placebo during the period ahead of surgery. Colorectal biopsy samples and blood samples were obtained from the patients before and after treatment and were analyzed for *p53* expression and serum TNF-α levels respectively. A significant reduction in the serum levels of TNF-α was observed in the patients treated with curcumin, whereas no such effect was observed in the placebo group. In the same way, the number of apoptotic cells was increased after treatment with curcumin compared to baseline values, whereas no significant change was observed in the placebo group. Moreover, treatment with curcumin increased the expression of *p53* and Bax and inhibited expression of Bcl-2 in the colorectal tissue [132].

More recently, the safety and efficacy of curcumin in familial adenomatous polyposis was evaluated in a double-blinded randomized trial by Cruz-Correa et al. [136]. In this study, 44 patients with familial adenomatous polyposis with at least five intestinal adenomatous polyps who had not undergone colectomy were included in this trial and were randomly allocated to two groups receiving either pure curcumin (3 g per day orally) or placebo for 12 months. The main outcome measures were the number and size of lower gastrointestinal tract polyps, which were assessed every four months for one year. At the end of the study, no significant difference was found in the mean number or mean size of polyps between the curcumin group and the placebo group. The adverse effects were very few, and not significantly different from the placebo group. These results show the low efficacy but high safety of oral curcumin at the administered dose in patients with familial adenomatous polyposis [136].

#### 4.2.2. Pancreatic Cancer

The efficacy of curcumin in the treatment of pancreatic cancer was investigated in a nonrandomized, open-label, phase II clinical trial conducted by Dhillon et al. [137]. In this study, 25 patients with histologically confirmed pancreatic adenocarcinoma were treated with a combination of curcuminoids (curcumin, desmethoxycurcumin, and bisdesmethoxycurcumin), at a dose of 8 g per day for eight weeks. The patients did not receive any chemotherapy or radiotherapy from four weeks before commencing the trial and also during the trial. Tumor response (as per the classic Response Evaluation Criteria in Solid Tumors criteria), tumor markers, and serum cytokine levels were assessed after 8 weeks. In addition, the effect of orally administered curcumin on constitutive and tumor necrosis factor-α–induced binding expression of NF-κB, COX-2, and phosphorylated signal transducer and activator of transcription 3 (pSTAT3) in peripheral blood mononuclear cells pretherapy and on day 8 were determined, as well as curcumin pharmacokinetics.

Low steady-state levels of curcumin glucuronide and curcumin sulfate indicated poor oral bioavailability. As a result, only two patients showed a clinical biological response to curcumin therapy, and one other patient showed a brief tumor regression accompanied by a considerable increase in serum cytokine levels (IL-6, IL-8, IL-10, and IL-1 receptor antagonists). On the other hand, curcumin down-regulated the expression of NF-κB, COX-2, and pSTAT3 in peripheral blood mononuclear cells obtained from patients. No treatment-related toxic effects were reported in any patients [137]. 

In a more recent study by Epelbaum et al. [138], patients with either advanced local or metastatic pancreatic cancer were treated with a combination of curcumin (8 g/day per oral) and gemcitabine (1000 mg/m^2^ IV once per week) for three out of four weeks of each chemotherapy cycle. The primary outcome was time to tumor progression and the main secondary outcome was toxicity profile. In the study, eight out of seventeen patients were noncompliant with curcumin due to abdominal pain, five of which discontinued treatment before two weeks and three received adjusted doses of curcumin for the rest of the study (4 g/day). One patient died during the first cycle due to cardiac problems not associated with curcumin. One patient developed grade II neutropenia and one patient grade I thrombocytopenia. The time to tumor progression was between one and twelve months (median two months), and the overall survival time was between one and 24 months (median 6). Based on these results, they concluded that the combination therapy with curcumin and gemcitabine in pancreatic cancer is feasible; however, the dose of curcumin should be less than 8 g/day [138].

#### 4.2.3. Prostate Cancer

In a double-blinded, randomized, placebo-controlled trial by Hejazi et al. [142], the effect of curcumin on the oxidative status of patients with prostate cancer during radiotherapy was evaluated. In this study, 40 patients were included in the trial and were randomly assigned to receive either curcuminoids (curcumin, desmethoxycurcumin, and bisdesmethoxycurcumin, 3 g per day per oral route) or placebo prior to and during external-beam radiation therapy. The outcome measures for oxidative status were the plasma total antioxidant capacity (TAC), superoxide dismutase (SOD) activity, catalase activity, and glutathione peroxidase activity, three months after radiotherapy. In addition, the level of prostate specific antigen (PSA) was used as a measure of successful treatment.

A significant increase in TAC and a significant decrease in SOD activity was observed after radiotherapy compared to the baseline (pretreatment) values, suggesting an antioxidant effect of curcumin, whereas no significant changes were observed in catalase activity and glutathione peroxidase activity. The levels of PSA were significantly reduced compared to baseline levels in both groups, indicating successful treatment; nevertheless, there was no significant difference between the two groups, indicating that curcumin did not affect the efficacy of the radiotherapy [142].

The effect of a combination of curcumin and soy isoflavones on the expression of PSA in men with elevated levels of PSA (but neither prostate cancer nor prostatic intraepithelial neoplasia) was investigated in a randomized placebo-controlled double-blind study by Ide et al. [141]. A total of 85 patients were included in this study and were randomly assigned to receive either a supplement containing a combination of isoflavones and curcumin or placebo. Systematic prostate biopsy was performed on the patients before and six months after treatment, and the levels of PSA were determined. The curcumin/isoflavone treatment considerably decreased the levels of PSA in the patients with an initial PSA ≥ 10 µg/mL as compared to the placebo group who did not show such change, which they attributed to the synergistic antiandrogen effect of curcumin and isoflavones [141]. However, since there was no comparison between the effects of treatment with isoflavones alone and curcumin alone compared to combination therapy on the levels of PSA, it is hard to accept the authors’ claim that the combination therapy has advantages over monotherapy as no such evidence is provided.

#### 4.2.4. Breast Cancer

Bayet-Robert et al. [128] evaluated the feasibility and tolerability of a combination of curcumin and docetaxel in 14 patients with metastatic or locoregionally recurrent advanced breast cancer in an open-label phase I dose escalation clinical trial. The patients received an I.V. infusion of Docetaxel (100 mg/m^2^) every three weeks for six chemotherapy cycles, and oral curcumin (starting from 500 mg/day and increased until a dose-limiting toxicity would occur) for seven consecutive days in each cycle (from five days before to two days after administration of docetaxel). The primary endpoint was the maximal tolerated dose of curcumin when administered in combination with a standard dose of docetaxel in the patients. Secondary outcomes were toxicity, safety, and clinical response to the combination therapy, as well as levels of CEA tumor marker and vascular endothelial growth factor (VEGF) as a positive endogenous modulator of angiogenesis.

The maximal tolerated dose of curcumin was found to be 8 g/day as in higher doses, dose-limiting toxicities (neutropenia, anemia, and severe diarrhea) were observed, leading to the discontinuation of the trial in two patients. Other toxicities (oral cavity mucositis, hand-foot syndrome, nail changes, dermal changes, conjunctivitis, and fatigue) were either not persistent or were treated easily so did not affect the continuation of the trial. However, due to noncompliance of a number of patients with the doses higher than 6 g/day, in the end, this dose was recommended as the maximal tolerated dose to be considered for phase II clinical trials. In terms of clinical and biological response (decrease in CEA tumor marker across the treatment and regression of nonmeasurable lesions), some degree of improvement was observed in most patients, with five patients showing a partial response to treatment and three patients having stable disease at least six weeks after the last cycle of treatment. No disease progression was observed in any of the patients. Moreover, curcumin/docetaxel combination significantly decreased the levels of VEGF after three cycles of treatment [128].

#### 4.2.5. Head and Neck Cancer

Kim et al. [135] performed a pilot study in patients with head and neck squamous cell carcinoma (HNSCC) to determine the effect of curcumin on inhibiting IκB kinase β (IκKβ) activity and suppressing the proinflammatory cytokines. The patients were asked to chew curcumin tablets (2 mg), their saliva samples were collected before and after chewing the tablets, and the IκKβ activity was measured, as well as the levels of salivary cytokines interleukin (IL)-6 and IL-8. Curcumin resulted in a reduction in IκKβ activity in the salivary cells of HNSCC patients. There was a brief reduction in IL-8 expression in eight of 21 post-curcumin samples; however, this reduction was not statistically significant. On the other hand, there was a marked decrease in the expression of other cytokines, including IL-10, IFN-γ, IL-12p70, and IL-2 clustered together, and also granulocyte macrophage colony stimulating factor (GMCSF) and TNF-α clustered together. These results show the inhibitory effect of curcumin on IκKβ activity in the salivary cells of patients with HNSCC; therefore, they suggested considering IκKβ as a biomarker for detecting the effect of curcumin in head and neck cancer [135]. 

## 5. Conclusions and Future Perspectives

Curcumin, the active ingredient of the *Curcuma longa* extract, has been studied widely over the past few decades for its anti-inflammatory, antioxidant, anticancer, and antiandrogenic effects. Curcumin has shown considerable anticancer effects against several different types of cancer, including prostate cancer, breast cancer, colorectal cancer, pancreatic cancer, and head and neck cancer both in vitro and in vivo. Furthermore, its efficacy and safety in cancer patients either alone or in combination with other anticancer agents has been proven in several clinical studies with human subjects. Curcumin is believed to exert its anticancer activity via multiple mechanisms, interfering with different cellular pathways and inducing/inhibiting the production of various types of cytokines, enzymes or growth factors such as MAPK, EGF, NFκB, PKD1, COX-2, STAT3, TNF-α, and IκKβ. However, the anticancer application of curcumin has been limited mainly due to its low water solubility, which results in low cellular uptake and poor oral bioavailability, as well as low chemical stability. In order to overcome these limitations, different approaches have been made, such as structural modification and the use of drug delivery systems. The key pharmacophores contributing to the biological activity of curcumin are known to be the hydrogen donor group, the β-diketone moiety, the phenyl rings, and the substituent groups on them. Chemical modification of these moieties has led to curcumin derivatives with higher efficacy and/or enhanced water solubility or stability. In addition, various types of delivery systems have been developed for curcumin delivery to cancer cells or animal xenografts using a variety of natural or synthetic polymers, lipids, or proteins, some of which have improved the stability and/or cellular uptake of curcumin, thus giving rise to a stronger anticancer response.

In spite of the tremendous effort to improve the physicochemical and biological properties of curcumin, there are still several issues to be addressed in regard to its bioavailability, potency, and specificity for the target tissue. The medicinal chemistry approaches to improving the pharmacological properties of curcumin have not managed to increase its potency significantly, and the curcumin derivatives are not more potent than curcumin itself. Due to the low potency of curcumin and its derivatives, higher doses are required to see a therapeutic response, which increases the adverse effects and reduces the patient compliance. Another drawback of the structural modification is that it is difficult to achieve a balance between efficacy and solubility, and in most cases, one has been sacrificed in favor of the other. Most of the structural modifications that improve curcumin efficacy make the molecule more hydrophobic and reduce its solubility. Therefore, more work has to be done in this regard to overcome this problem. Although various types of drug delivery systems have been used to enhance the cellular uptake and efficacy of curcumin, most of these formulations have remained at the proof of concept level and have not been evaluated in clinical trials. There is a lack of clinical studies to evaluate the safety and efficacy of these curcumin delivery systems in humans before they can find their way to the pharmaceutical market. Moreover, most of the currently developed drug delivery systems for curcumin lack specificity for the target tissue. Hence, there is still much room for improvement in the curcumin delivery systems in terms of selectivity for specific tumor tissues. Tissue-specific curcumin delivery enhances the local drug concentrations in the site of action and therefore results in higher efficacy (with lower doses of curcumin) and less adverse effects. 

## Figures and Tables

**Table 1:** Changes to the pharmacological activity of curcumin derivatives compared to curcumin.

Curcumin Derivative	Chemical Modification	Activities	References
Dimethyl curcumin (ASC-J9)	Methyl groups substitution on R2 and R4	Enhanced activity toward prostate and breast cancer	[20,21,22,38]
Vanadium, gallium, and indium complexes	Metal complexation by the β-diketones	Enhanced cytotoxic activity	[39]
Tetrahydrocurcumin (THC)	Hydrogenated diketone moiety	Enhanced antioxidant activity but loss of DNA binding and STAT3 ^a^ inhibition properties	[31,33]
Modified aromatic rings curcumin compounds	Introduction of cyclohexane bridge	Improved mitochondrial membrane permeability during lymphoma therapy	[40]
Metallo-curcumin (Cu^2+^/Ni^2+^/Zn^2+^)	Metal complexation by the β-diketones	Enhanced water-solubility and improved DNA binding	[34]
Glycosylated curcumin derivative	Glycol groups substitution on the aromatic rings	Higher potency, aqueous solubility, and chelating properties	[41]
Cu^2+^ conjugate of synthetic curcumin analogues	Conjugation reaction on the keto-enol moiety	Stronger inhibition of TNF ^b^-induced NF-κB ^c^ activation in leukemic KBM-5 cells	[42]
Cyclic curcumin derivatives	Boron trioxide-mediated aldol condensation	Enhanced cytostatic, antitumor, and antioxidant activity	[43]
Curcumin carbocyclic analogues	Introducing carboxyl group at the diketone moiety	Enhanced antioxidant activity and stronger inhibition of HIV ^d^ 1 protease	[44]
Hydrazinocurcumin	Replacing the diketone moiety with hydrazine derivative	Higher efficacy in inhibition of colon cancer progression via antagonism of Ca^2+^/CaM ^e^ function	[45,46]
Semicarbazone	Introducing NNHCONH_2_ at the keto-enol moiety	Enhanced antioxidant, antiradical, and antiproliferative activity	[47]

^a^ STAT3: signal transducer and activator of transcription 3; ^b^ TNF: Tumor necrosis factor; ^c^ NF-κB: nuclear factor κ-light-chain-enhancer of activated B cells; ^d^ HIV: Human immunodeficiency virus; ^e^ Ca^2+^/CaM: calcium/calmodulin.

**Table 2:** Examples of recent curcumin delivery systems.

Nanoformulation	Particle Size	Application	Outcome	Reference
Curcumin-loaded liposomal PMSA ^a^ antibodies	100–150 nm	Human prostate cancer (LNCa, C4-2B)	Enhanced antiproliferative efficacy and targeting	[63]
Curcumin-loaded magnetic silk nanoparticles	100–350 nm	Human breast cancer (MDA-MB-231) cells	Enhanced cellular uptake and growth inhibition	[54]
Curcumin/MPEG ^b^-PCL ^c^ micelles	27 ± 1.3 nm	Colon carcinoma (C-26) cells	Enhanced cancer growth inhibition	[64]
Curcumin nanoemulsion	<200 nm	Human ovarian adenocarcinoma cells (SKV3)	Increased cytotoxicity	[65]
Curcumin loaded liposomes coated with N-dodecyl chitosan-HPTMA ^d^ chloride	73 nm	Murine fibroblasts (NIH3T3) and murine melanoma (B16F10) cells	Specific toxicity in murine melanoma (but not in fibroblasts)	[66]
Curcumin-PLGA ^e^ nanoparticles	248 ± 1.6 nm	Erythroleukemia type 562 cells	Improved clinical management of leukemia	[65]
Curcumin loaded lipo-PEG ^f^-PEI ^g^ complexes	269 nm	Melanoma (B16F10) and colon carcinoma (CT-26) cells	Increased cytotoxicity	[67]
Curcumin–chitosan nanoparticles	100–250 nm	Melanomas	Enhanced antitumor effect	[68]
ApoE ^h^ peptide-functionalized curcumin-loaded liposomes	132 nm	RBE4 cell monolayer	Increased accumulation in brain capillary endothelium	[69]
Curcumin-crosslinked polymeric Nanogels	10–200 nm	Breast and pancreatic cancers	Higher stability and enhanced antitumor effect	[70]
Curcumin-loaded chitin nanogels	70–80 nm	Human skin melanoma (A385) and human dermal fibroblasts (HDF)	Specific toxicity in skin melanoma (lower toxicity in HDF)	[70]
Curcumin-loaded lipid-core nanocapsules	196 ± 1.4 nm	Rat C6 and U251MG glioma cell lines	Decreased tumor size and prolonged survival	[71]
Liposome-encapsulated curcumin	Not reported	Head and neck squamous cell carcinoma (HNSCC) cell lines (CAL27 and UM-SCC1)	Cancer growth suppression both in vitro and in vivo	[72]

^a^ PMSA: Prostate membrane specific antigen; ^b^ MPEG: Monomethoxy poly ethylene glycol; ^c^ PCL: Poly(ε-caprolactone); ^d^ HPTMA: N-[(2-hydroxy-3-trimethylamine) propyl; ^e^ PLGA: Polylactic-co-glycolic acid; ^f^ PEG: Poly ethylene glycol; ^g^ PEI: Polyethyleneimine; ^h^ ApoE: Apolipoprotein E.

**Table 3:** Clinical studies of curcumin in the prevention/treatment of different types of cancer.

Type of Cancer	Type of Study	No of Patients	Dose of Curcumin	Endpoints	Results	References
BPH ^a^	Pilot product evaluation study	61	1g/day for 24 weeks	Signs and symptoms, quality of life	Reduced signs and symptoms, improved quality of life	[127]
Breast	Phase I clinical trial	14	0.5–8 g/day for 7 days plus docetaxel	Maximal tolerated dose of curcumin, toxicity, safety, efficacy, levels of VEGF ^b^ and tumor markers	No cancer progression, partial response in some patients, low frequency of toxic effects, decreased levels of VEGF	[128]
CML ^c^	Randomized controlled trial	50	5 g TID ^d^ for 6 weeks plus imatinib (400 mg BD ^e^)	Plasma nitric oxide levels	Reduced nitric oxide levels	[129]
Colorectal	dose-escalation pilot study	15	40–200 mg/day for 29 days	Blood COX-2 ^f^ activity and PGE2 ^g^ levels	Dose-dependent decrease in PGE2 levels	[130]
	Phase I does-escalation trial	15	0.45–3.6 g/day for 4 months	Levels of curcumin and its metabolites in plasma urine, and feces; levels of PGE2 and glutathione *S*-transferase activity in blood	Dose-dependent decrease in PGE2 levels, low concentrations of curcumin and its metabolites in plasma and urine	[131]
	Phase I does-escalation trial	12	0.45 g, 1.8 g, 3.6 g per day for 7 days	Concentration of curcumin and its metabolites in plasma and colorectal tissue	Biologically active concentrations of curcumin in the colorectal tissue	[105]
	Phase I clinical trial	126	360 mg TID for 10–30 days	Serum levels of TNF-α ^h^, *p53* expression in tumor tissue	Decreased serum levels of TNF-α, increased expression of *p53* in colorectal tissue	[132]
	Phase II clinical trial	44	2 g/day and 4 g/day for 1 month	Concentration of PGE2 and 5-HETE ^i^ within ACF ^j^ and normal mucosa, total ACF number	Reduced number of ACF with dose of 4 g	[133]
	Pilot study	26	2.35 g/day for 14 days	Safety, tolerance, levels of curcumin in colonic mucosa	Safe and well tolerated, Prolonged biologically active levels of curcumin achieved in colon tissue	[134]
HNSCC ^k^	Pilot study	21	1 g single dose	IκKβ ^l^ kinase activity, cytokine levels in saliva	Reduced IκKβ activity in the salivary cells	[135]
Intestinal Adenoma	Randomized controlled trial	44	1.5 g BID for 12 months	total number of polyps, mean polyp size, adverse effects	No significant clinical response, very few adverse effects	[136]
Pancreatic	Phase II clinical trial	25	8 g/day for 8 weeks	Tumor response, tumor markers, adverse effects	Poor oral bioavailability, biological response in only 2 patients, no toxicities	[137]
	Phase II clinical trial	17	8 g/day for 4 weeks	Time to tumor progression (TTP) and toxicity profile	TTP of 1–12 months (median 2 months), high frequency of side effects	[138]
	Phase I/II clinical trial	21	8 g/day for 14 days plus gemcitabine	patient compliance, toxicity, efficacy	Safe and well tolerated, median overall survival time of 161 days	[139]
	Phase I clinical trial	16	200–400 mg/day for 9 months	Safety, pharmacokinetics, NF-κB ^m^ activity, cytokine levels, efficacy and quality of life	Safe, highly bioavailable, no significant changes in NF-κB activity or cytokine levels, improved quality of life	[140]
Prostate	Randomized controlled trial	85	100 mg plus 40 mg soy isoflavones for 6 months	Serum PSA ^n^ levels	Decreased levels of PSA in patients with an initial PSA ≥ 10 µg/mL	[141]
	Randomized controlled trial	40	3 g/day for 3 months as a supplement to radiotherapy	biochemical and clinical progression-free survivals, alterations in the activity of antioxidant enzymes	Considerable antioxidant effect, decreased levels of PSA	[142]
Solid tumors	Randomized controlled trial	80	180 mg/day for 8 weeks	Changes in quality of life, serum levels of inflammatory mediators	Improved quality of life, reduced levels of inflammatory mediators	[143]

^a^ BPH: benign prostatic hypertrophy; ^b^ VEGF: vascular endothelial growth factor; ^c^ CML: chronic myeloid leukemia; ^d^ TID: three times daily; ^e^ BD: Twice daily; ^f^ COX-2: cyclooxygenase-2; ^g^ PGE2: Prostaglandin E2; ^h^ TNF-α: tumor necrosis factor α; ^i^ 5-HETE: 5-hydroxyeicosatetraenoic acid; ^j^ ACF: aberrant crypt foci; ^k^ HNSCC: Head and neck squamous cell carcinoma; ^l^ IκKβ: IκB kinase β; ^m^ NF-κB: Nuclear factor κB; ^n^ PSA: prostate-specific antigen.
